# Editorial

**DOI:** 10.1007/s00414-020-02442-6

**Published:** 2020-10-17

**Authors:** Thomas Bajanowski

**Affiliations:** Institute of Legal Medicine, University Hospital Essen, Hufelandstr. 55, 45122 Essen, Germany

Dear colleagues, dear editors, dear reviewers, dear authors,

After more than 20 years of work with the International Journal of Legal Medicine, it is time for me to relinquish my role as Editor-in-Chief. Therefore, I would like to take this opportunity to thank you all for your trusting cooperation over the previous years. Without the commitment of all of you it would not have been possible to develop our journal as successfully as it is. It was always a pleasure for me to interact with authors and reviewers and to have the opportunity to accompany a high number of excellent manuscripts on their way to publication. I would like to thank Prof. Pfeiffer and Prof. Schmeling as well as Mrs. Beckhoff for years of trusting and friendly collaboration. Furthermore, I wish to thank all the friends from the presidium of the International Academy of Legal Medicine, in particularly the past president, Prof. Davide Ferrara, and the actual president, Prof. Angel Carracedo. I have really enjoyed working with you. Finally yet importantly, I would like to thank the Springer representatives who are/were responsible for the Journal from the publisher site. Dr. Pillmann an now Mrs. Ullmann supported the editors in all important organizational questions and had/has a significant influence on the expansion of the content and scope of the journal.

At the beginning of next year, Prof. Schmeling will follow me as Editor-in-Chief, and my colleague and friend Prof. Tony Fracasso will follow me as associated editor who is responsible for morphology and clinical forensic medicine. As you all know, both are extraordinary scientists, one is a specialist in anthropology and the other in forensic morphology. I am sure that Prof. Fracasso, the new member of the team will support the editorial team by excellent work. I wish him all the best for this new task.

Best wishes to all of you and for the journal.

Thomas Bajanowski

